# Development and validation of a prediction score for failure to casirivimab/imdevimab in hospitalized patients with COVID-19 pneumonia

**DOI:** 10.3389/fmed.2024.1293431

**Published:** 2024-03-11

**Authors:** Alessandro Cozzi-Lepri, Vanni Borghi, Salvatore Rotundo, Bianca Mariani, Anna Ferrari, Cosmo Del Borgo, Francesca Bai, Pietro Colletti, Piermauro Miraglia, Carlo Torti, Anna Maria Cattelan, Giovanni Cenderello, Marco Berruti, Carlo Tascini, Giustino Parruti, Simona Coladonato, Andrea Gori, Giulia Marchetti, Miriam Lichtner, Luigi Coppola, Chiara Sorace, Alessandra D'Abramo, Valentina Mazzotta, Giovanni Guaraldi, Erica Franceschini, Marianna Meschiari, Loredana Sarmati, Andrea Antinori, Emanuele Nicastri, Cristina Mussini

**Affiliations:** ^1^Centre for Clinical Research, Epidemiology, Modelling and Evaluation (CREME), Institute for Global Health, University College London, London, United Kingdom; ^2^Clinic of Infectious Diseases, Azienda Ospedaliero-Universitaria Policlinico, Modena, Italy; ^3^Unit of Infectious and Tropical Diseases, University Magna Græcia, Catanzaro, Italy; ^4^Unità Organizzativa Complessa Malattie Infettive, Fondazione Istituto di Ricovero e Cura a Caratetre Scientifico, Cà Granda Ospedale Maggiore Policlinico, Milan, Italy; ^5^Unità Organizzativa Complessa Malattie Infettive, Azienda Ospedale Università, Padova, Italy; ^6^Unità Organizzativa Complessa Malattie Infettive, Ospedale SM Goretti, Latina, Italy; ^7^Clinica delle Malattie Infettive, Dipartimento di Scienze della Salute, ASST Santi Paolo e Carlo, University of Milano, Milan, Italy; ^8^Unit of Infectious Diseases, Paolo Borsellino Hospital, ASP Trapani, Trapani, Italy; ^9^SC Malattie Infettive Asl1 Imperiese, Imperia, Italy; ^10^Clinic of Infectious Diseases, University of Udine, Udine, Italy; ^11^Unit of Infectious Diseases, Hospital of Pescara, Pescara, Italy; ^12^Infectious Disease Unit, Ospedale L. Sacco, University of Milano, Milan, Italy; ^13^Clinical Infectious Diseases, Tor Vergata University, Rome, Italy; ^14^Istituto Nazionale Malattie Infettive L. Spallanzani, Rome, Italy; ^15^Department of Surgical, Medical, Dental and Morphological Sciences, University of Modena and Reggio Emilia, Modena, Emilia-Romagna, Italy

**Keywords:** casirivimab/imdevimab, COVID-19, mechanical ventilation, mortality, prediction score, SARS-CoV-2

## Abstract

**Introduction:**

Casirivimab and imdevimab (CAS/IMV) are two non-competing, high-affinity human IgG1 anti-SARS-CoV-2 monoclonal antibodies, that showed a survival benefit in seronegative hospitalized patients with COVID-19. This study aimed to estimate the day-28 risk of mechanical ventilation (MV) and death in individuals hospitalized for severe COVID-19 pneumonia and receiving CAS/IMV. Additionally, it aimed to identify variables measured at the time of hospital admission that could predict these outcomes and derive a prediction algorithm.

**Methods:**

This is a retrospective, observational cohort study conducted in 12 hospitals in Italy. Adult patients who were consecutively hospitalized from November 2021 to February 2022 receiving CAS/IMV were included. A multivariable logistic regression model was used to identify predictors of MV or death by day 28 from treatment initiation, and β-coefficients from the model were used to develop a risk score that was derived by means of leave-one-out internal cross-validation (CV), external CV, and calibration. Secondary outcome was mortality.

**Results:**

A total of 480 hospitalized patients in the training set and 157 patients in the test set were included. By day 28, 36 participants (8%) underwent MV and 28 died (6%) for a total of 58 participants (12%) experiencing the composite primary endpoint. In multivariable analysis, four factors [age, PaO^2^/FiO^2^ ratio, lactate dehydrogenase (LDH), and platelets] were independently associated with the risk of MV/death and were used to generate the proposed risk score. The accuracy of the score in the area under the curve (AUC) was 0.80 and 0.77 in internal validation and test for the composite endpoint and 0.87 and 0.86 for death, respectively. The model also appeared to be well calibrated with the raw data.

**Conclusion:**

The mortality risk reported in our study was lower than that previously reported. Although CAS/IMV is no longer used, our score might help in identifying which patients are not likely to benefit from monoclonal antibodies and may require alternative interventions.

## Introduction

The treatment of hospitalized patients with COVID-19 has undergone profound changes since March 2020, and interventions varied over time as a result of accumulating evidence from both observational and randomized studies conducted across different waves of the pandemic (1–3). This is particularly true for monoclonal antibodies. Since the beginning of the pandemic, it was understood that they could play a role in treating COVID-19 disease especially during the viral phase of the infection, as proved to be effective in animal models and *in vitro* studies on rapid culture conversion of SARS-CoV-2 infection (4, 5). Indeed, several monoclonal antibodies, when tailored against the specific predominant circulating viral variant, were shown to be effective in preventing SARS-CoV-2 infection (especially in immunocompromised individuals) and hospitalization if administered within the first 5–7 days after the infection (6–10).

Concerning hospitalized patients, since patients in this setting are typically in the inflammatory phase of COVID-19, the role of these compounds is more unclear. Indeed, two placebo-controlled randomized trials, one with bamlanivimab and one with either sotrovimab or the combination of BRII-196 and BRII-198, were terminated earlier due to futility (11–13).

Casirivimab and imdevimab (CAS/IMV) are two non-competing, high-affinity human IgG1 anti-SARS-CoV-2 monoclonal antibodies, which bind specifically to the receptor-binding domain of the spike glycoprotein of SARS-CoV-2, thereby blocking viral entry into host cells (14). Recently, two randomized trials were conducted among hospitalized patients with CAS/IMV: the RECOVERY platform trial and a smaller trial conducted in the USA on 1,336 patients (6, 15). The RECOVERY trial showed a clear benefit in terms of survival, particularly when restricting the analysis to patients with a negative antibody status at hospital admission (RR 0.79, 95% CI 0.69–0.91; *P* = 0.0009). A similar result in terms of survival, still when restricting the analysis to seronegative patients, was obtained in another recently published study (15). Based on these results, all international guidelines suggested monoclonal antibodies for hospitalized subjects seronegative for SARS-CoV-2 in the pre-Omicron era.

To our knowledge, no real-life data on CAS/IMV outcomes in hospitalized patients with severe COVID-19 pneumonia have been published until now. With these premises, the aim of our analysis was twofold: first, we aimed to estimate the in-hospital day-28 risk of mechanical ventilation (MV) and death in a large cohort of patients admitted to hospital with SARS-CoV-2 pre-omicron variants of concern (VoCs) and treated with CAS/IMV. Second, we aimed at identifying variables measured at the time of hospital admission that could predict these poor outcomes as well as derive a prediction algorithm for clinical use (16–18).

## Methods

This multi-center observational study analyzed individuals who confirmed positive for SARS-CoV-2 through nasal swab tests and hospitalized patients with severe COVID-19 who were treated with CAS/IMV in 12 tertiary care hospitals in Italy. Among the 12 hospitals, nine hospitals provided data for the training set and three hospitals provided for the test set (Supplementary Table S3). All adult patients consecutively hospitalized with severe COVID-19 and treated with CAS/IMV from November 2021 to March 2022 were included. Severe COVID-19 was defined in accordance with the NIH (National Institute of Health) guidelines. We excluded patients for whom there was no confirmation of the SARS-CoV-2 positive test, those who were not eligible for treatment with CAS/IMV, and those who started other treatments. The retrospective data were fully anonymized and were last assessed on February 2023. CAS/IMV were administered intravenously at the dose of 4 g for each antibody.

### IRB approval and data information

The study was approved by the ethics committee of each participating site.

### Data collection

The participants' demographics (age, sex, calendar month and year of hospitalization, vaccination history, and SARS CoV-2 serology test results), comorbidities, and laboratory and respiratory function parameters were collected at the time of hospital admission. Data were collected from both paper and electronic clinical records using a standardized operating procedure across clinics.

### Study outcomes

The primary outcome was the composite event of initiation of MV or death by day 28 from the date of starting CAS/IMV while the secondary outcome was death. Participants who experienced the event (MV or death) during the follow-up period and up to day 28 from starting the drug were labeled as events and the remaining were labeled as event-free.

### Statistical analysis

Participants' characteristics were described and compared based on their event/event-free status. For continuous variables (such as age and laboratory and respiratory function parameters), the ranks of the distributions were compared between events and event-free using the Mann–Whitney test. Chi-squared and Fisher exact tests were used to compare proportions as appropriate.

Factors that were most strongly associated with the primary outcome in univariable analysis were selected as candidates to be included in a prediction score. Univariable and multivariable associations between these factors and the risk of the outcome were estimated using the logistic regression method, and the magnitude of the association were expressed by means of odds ratios (OR) with 95% confidence interval.

For constructing the score for the continuous variables (16–18), participants were grouped according to the median value of these variables, and ORs of the primary endpoint associated with a value below/above the median were shown.

To evaluate the ability of the model to predict the outcomes, the area under the receiver operating characteristic (ROC) curve was calculated. This was first calculated in the training set and compared to the classifier with area under the curve (AUC) = 0.5 using a Mann-Whitney statistics and chi-square test. To control for extra-sample variation, a leave-one-out cross-validation (CV) was implemented. This means that N separate times, the function approximator was trained on all the data after excluding one participant and a prediction was made for this excluded person. The AUC in CV was computed and used to evaluate the predictive ability of the model by comparing it with the value obtained on the training set. In addition to evaluating the predictive ability, we also investigated the model calibration by means of a calibration plot in the test dataset.

Furthermore, we used an independent sample of patients obtained from other three Italian hospitals (one for each region of the country: North, Central, and South) with identical inclusion criteria and definition of outcomes (Supplementary Table S3, the test set).

Symmetrical analyses were performed for the primary and secondary endpoint of death. For the purpose of simplicity and to broadly categorize the risk of failing CAS/IMV, the study population was divided into those with low (0–5%), moderate (6–19%), and high (≥20%) risk of day-28 mechanical ventilation/death. These groups were matched to actual risk ranges calculated from the propensity score formula below Equation (1). Exact individual risk can be calculated *as per* the formula given below by entering the individual's own demographics and biomarkers values.


(1) $$\begin{array}{l} \text{Prob }\left(\text{MV}/\text{death}\right)=\theta /\left(1+\theta \right)\text{ where }\theta \\ \;=\text{ exp}\left(\beta 0\;+\;\beta 1\text{X}1\;+\;\beta 2\text{X}2\right), \end{array}$$

*X*1, *X*2, *etc*. are the patients' characteristic values at hospital admission, and β0, β1, β2, etc. are the parameter estimates from the logistic regression model.

The results were reported according to the TRIPOD guidelines (17, 18).

## Results

### Training set

The training set included a total of 480 hospitalized patients. By day 28 since the date of CAS/IMV initiation, 36 participants (8%) underwent MV and 28 died (6%) for a total of 58 participants (12%) who experienced the composite primary endpoint.

Table 1 shows the main demographic characteristics and average marker values recorded at hospital admission stratified by events/event-free status. As expected, the level of PaO^2^/FiO^2^ ratio and platelets were lower in events compared to event-free. In contrast, lactate dehydrogenase (LDH) levels were higher in events who were also more than 10 years older than event-free (72 years vs. 60 years on average). Table 2 shows the prevalence of comorbidities in events and event-free. Unexpectedly, the only condition strongly associated with the outcome was cardiac insufficiency (21% in events vs. 8% in event-free, *P* < 0.0001) which remained associated after controlling for age (Table 3).

**Table 1:** Baseline characteristics of the participants included in the training set by events/event-free status.

	**Event/Event-free status (Training dataset)**			
**Markers**	**MV/death**	**Event-free**	***p*** **-value** ^*^	**Total**
	***N** =* **58**	***N** =* **422**		***N** =* **480**
Female, *n* (%)	21 (36.2)	183 (43.4)	0.302	204 (42.5)
Age, years mean (SD)	70 (16)	62 (17)	0.001	63 (17)
Charlson Index, mean (SD)	2 (2)	1 (2)	0.184	1 (2)
BMI, mean (SD)	28 (6)	27 (5)	0.457	27 (5)
Admitted in 2021, *n* (%)	47 (81.0)	380 (90.0)	0.040	427 (89.0)
Previous vaccination, *n* (%)	13 (22.8)	88 (22.6)	0.967	101 (22.6)
Positive COVID-19 serology, *n* (%)	9 (21.4)	65 (20.3)	0.866	74 (20.4)
PO_2_/FiO_2_, mean (SD)	231.4 (89.3)	309.9 (83.1)	< 0.001	309.9 (83.1)
White cells, mean (SD)	5964 (4219)	6009 (8879)	0.970	6003 (8449)
IL-6, pg/ml mean (SD)	396.9 (920.7)	196.3 (722.4)	0.160	232.5 (763.2)
LDH, mg/dl mean (SD)	617.7 (427.8)	384.3 (230.6)	< 0.001	412.5 (272.6)
CRP, mg/dl mean (SD)	4.7 (6.2)	4.2 (7.1)	0.600	4.3 (7.0)
Tot Lymphocytes, mg/dl mean (SD)	1306 (3128)	1644 (8600)	0.767	1603 (8129)
Platelets, mg/dl mean (SD)	161.0 (77.9)	197.6 (91.7)	0.004	193.1 (90.9)

N, number; LDH, lactate dehydrogenase; IL-6, interleukin-6; CRP, C-reactive protein; BMI, body mass index; MV, mechanical ventilation; SD, standard deviation. ^*^Chi2 for gender and unpaired *t*-test.

**Table 2:** Comorbidities at hospital admission in the participants included in the training set by events/event-free status.

	**Events/event-free status (REG score)**			
**Comorbidities**	**MV/death**	**Event-free**	***p*** **-value** ^*^	**Total**
	***N** =* **58**	***N** =* **422**		***N** =* **480**
**Conditions, n (%)**				
BMI >30	14 (26.9)	89 (25.9)	0.872	103 (26.0)
AIDS	0 (0.0)	1 (0.2)	0.711	1 (0.2)
Cardio-ischemia	8 (13.8)	41 (9.7)	0.337	49 (10.2)
Cerebrovascular disease	4 (6.9)	26 (6.2)	0.828	30 (6.3)
Connective	4 (6.9)	17 (4.0)	0.317	21 (4.4)
Dementia	4 (6.9)	24 (5.7)	0.713	28 (5.8)
Diabetes	11 (19.0)	64 (15.2)	0.455	75 (15.6)
Hematologic cancer	2 (3.4)	35 (8.3)	0.195	37 (7.7)
Hemiplegia	1 (1.7)	8 (1.9)	0.928	9 (1.9)
Liver disease	3 (5.2)	16 (3.8)	0.613	19 (4.0)
Cardiac insufficiency	12 (20.7)	32 (7.6)	0.001	44 (9.2)
Pulmonary disease	8 (13.8)	50 (11.8)	0.670	58 (12.1)
Renal disease	6 (10.3)	28 (6.6)	0.302	34 (7.1)
Solid cancer	8 (13.8)	36 (8.5)	0.193	44 (9.2)
Gastric ulcer	2 (3.4)	12 (2.8)	0.798	14 (2.9)
Vasculopathy	6 (10.3)	21 (5.0)	0.096	27 (5.6)

^*^Chi-squared test. BMI, body mass index; MV, mechanical ventilation; N, number.

**Table 3:** Odds ratio of MV/death from fitting a logistic regression model (Training set).

	**OR of MV/death (Training dataset)**					
	**Unadjusted OR (95% CI)**	***p*** **-value**	**Adjusted1** ^*^ **OR (95% CI)**	***p-*** **value**	**Adjusted2**^&^ **OR (95% CI)**	***p*** **-value**
PO_2_/FiO_2_, per log10 lower	461.5 (69.67, 3057)	< 0.001	487.2 (68.42, 3470)	< 0.001	317.7 (32.04, 3151)	< 0.001
IL-6, per log10 mg/dl higher	2.51 (1.53, 4.12)	< 0.001	2.60 (1.55, 4.37)	< 0.001		
LDH, per log10 mg/dl higher	20.54 (6.52, 64.74)	< 0.001	27.20 (8.16, 90.62)	< 0.001	11.20 (2.58, 48.65)	0.001
Platelets, per log10 mg/dl lower	9.76 (2.69, 35.36)	< 0.001	7.42 (2.00, 27.58)	0.003	12.91 (2.41, 69.23)	0.003
White Cells, per log10 higher	1.27 (0.43, 3.77)	0.662	1.17 (0.39, 3.51)	0.785		
Lymphocytes, per log10 higher	3.63 (1.31, 10.04)	0.013	2.72 (0.97, 7.63)	0.058	1.19 (0.41, 3.43)	0.745
CRP, per log10 higher	1.28 (0.93, 1.76)	0.137	1.37 (0.95, 1.96)	0.090	1.11 (0.75, 1.65)	0.611
Age, per 10 years older	1.32 (1.11, 1.57)	0.002				
Charlson Index, per 1 unit higher	1.09 (0.96, 1.24)	0.186				
Female vs. male	0.74 (0.42, 1.31)	0.302				
Cardiac insufficiency	3.18 (1.53, 6.60)	0.002	2.22 (1.02, 4.81)	0.002	1.21 (0.46, 3.19)	0.699
Renal disease	1.62 (0.64, 4.11)	0.306	1.01 (0.38, 2.69)	0.306	0.78 (0.23, 2.63)	0.691
Vasculopathy	2.20 (0.85, 5.71)	0.104	1.53 (0.57, 4.10)	0.104	1.92 (0.61, 6.04)	0.267
BMI, >30 vs. ≤ 30	1.06 (0.55, 2.04)	0.872				
Year of hospital admission, 2021 vs. 2022	0.47 (0.23, 0.98)	0.044	0.60 (0.28, 1.27)	0.183	0.63 (0.25, 1.55)	0.313
At least 2 doses of vaccine, yes vs. no	1.01 (0.52, 1.97)	0.967				
SARS-CoV-2 serology, pos vs. neg	1.07 (0.49, 2.35)	0.866				

CRP, C-reactive protein; BMI, body mass index; IL-6, interleukin-6; MV, mechanical ventilation. ^*^adjusted for gender and age. ^&^adjusted for gender age and other markers shown in this table.

Table 3 shows factors significantly associated with the risk of the outcome in the univariable analysis. These included the following four factors: age, PaO^2^/FiO^2^ ratio, LDH, and platelets, which were considered to build the predictive score. Notably, PaO^2^/FiO^2^ ratio, LDH, and platelets remained significantly associated after mutual adjustment as well as after controlling for age. PaO^2^/FiO^2^ ratio that indicated the level of respiratory function at admission was by large the factor associated with the bigger effect (OR).

IL-6 was also strongly associated with the risk of MV/death but was left out of the score because too many participants had a missing value for this marker. In contrast, there was little evidence for an association between the extent of total comorbidities (summarized by the Charlson Index), year of hospital admission (2021 vs. 2022), vaccination history, and SARS-CoV-2 serology test results. Consequently, these factors were also left out of the score.

Calibration of the model in the test set was not perfect but lied close to the “perfect calibration” dotted line for the slope and within its 95% confidence limits (Supplementary Figure S1). The analysis for the secondary endpoint of death identified the same predictors, although the magnitude of the association was smaller for PaO^2^/FiO^2^ and LDH (Supplementary Table S2).

### Internal validation

**Figure 1:**
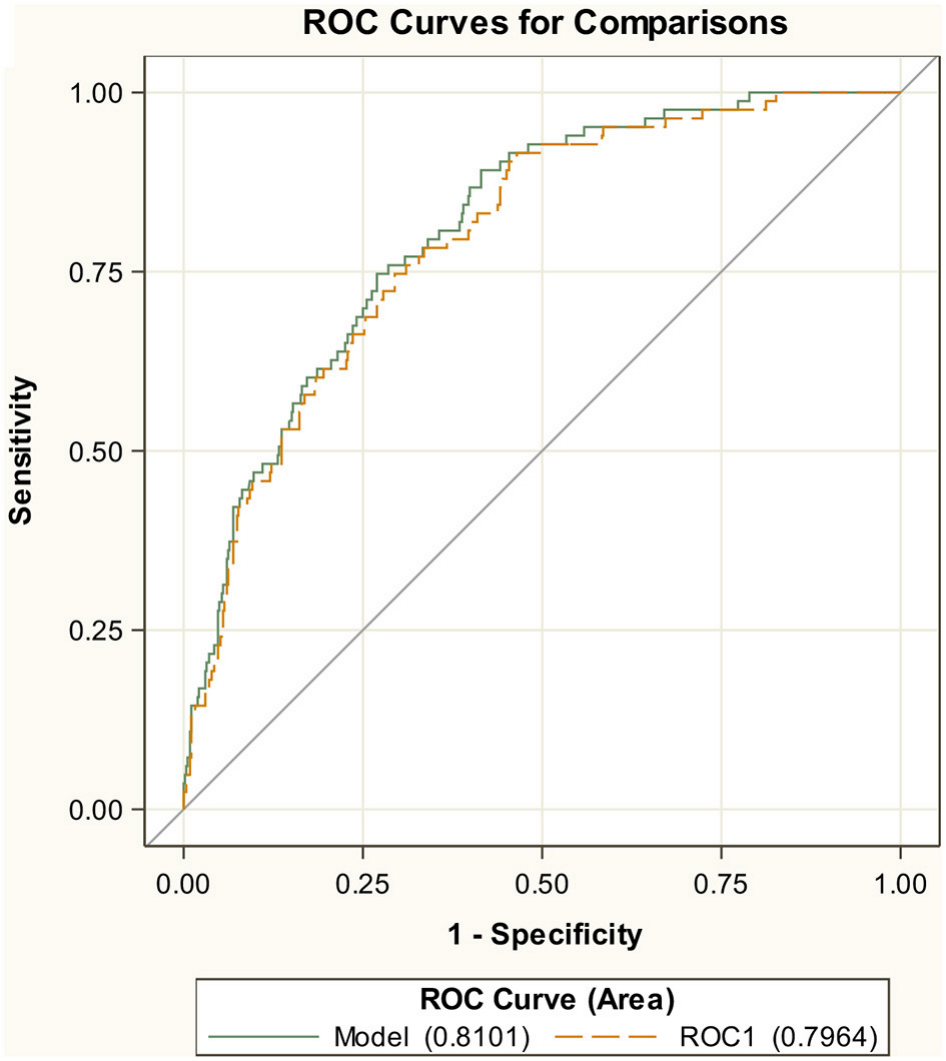
Internal cross-validation results. ROC Curve of AUC endpoint MV/death.

The AUC of the ROC curve using the training dataset was 0.81, indicating a good trade-off between sensitivity and specificity (Figure 1). The AUC was significantly better than a random classification of the participants (Mann–Whitney test vs. AUC = 0.5, *P* < 0.001). Although the performance of the model is likely to be overestimated in training, the internal validation, by means of CV, provided only a slightly lower value for AUC = 0.80 (Figure 1). For the secondary endpoint of death, the AUCs were even bigger with 0.87 in the training set and 0.85 in cross-validation (Supplementary Figure S2).

### External validation

**Figure 2:**
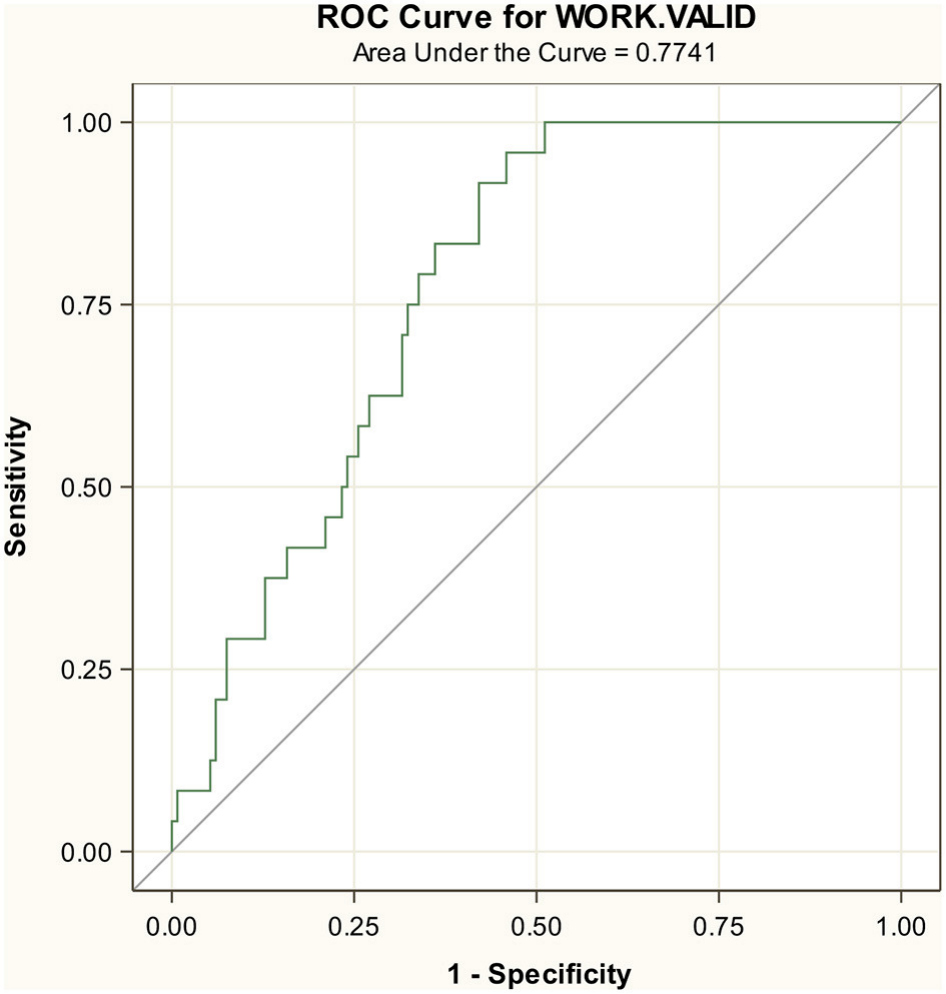
External cross-validation results. ROC Curve of AUC endpoint MV/death.

The test set included 157 participants with 24 events. In the test set, 44% were female participants, with the mean age of 69 years, PaO^2^/FiO^2^ ratio of 319 mmHg, LDH of 275.8 mg/dL, and platelets 189.4 mg/dL (Supplementary Table S1). The AUC in the test set was 0.77 for the composite endpoint (Figure 2) and slightly better for the endpoint death, indicating a smaller decrease in accuracy from 0.87 to 0.86 (Supplementary Figure S3).

### Derivation of the score for the primary endpoint MV/death

Based on the estimates of the logistic regression analysis, a simplified prediction score was developed from fitting the factors as binary variables (using the median as the threshold for continuous variables) and allocating weights to each of the four components of the score. Thus, for example, a virtual person aged 62+ with LDH above the median at hospital admission and PaO^2^/FiO^2^ ratio and platelets below the median has all four risk factors (age, LDH, PaO^2^/FiO^2^ ratio, and platelets, Table 4). Thus, for our virtual participant who is older than 62 years (+6), with a PaO^2^/FiO^2^ ratio < 306 mmHg (+8), LDH >287 (+2), and platelets < 177 (+4), his/her simplified sum risk score would be 6 + 7 + 3 + 5 = 21, placing him/her in the high-risk (≥20) category. The exact propensity risk for this person (i.e., his/her probability of experiencing MV/death given his/her covariate profile) estimated from the logistic regression model is 37.4% (it can be calculated from the Equation 1 described in the Methods and shown again at the bottom of Table 4).

**Table 4:** Risk scores for ICU/death on CAS/IMV: exact and simplified risk scores.

**(A)**						
**Characteristic**	**Coefficient of logistic regression**	**Simplified individual score**	**Example for a virtual participant** ^$^			
			**Observed characteristic**	**Contribution**	**Exact propensity score from the model**	**Total risk score**
**Age, years**						
18-61	0	0				
62+	+1.02	+6	**X**	+6		
**LDH**						
25-286	0	0				
287+	+0.51	+3	**X**	+3		
**PaO2/FiO2 ratio mmHg**						
306+	0	0				
78-305	+1.18	+7	**X**	+7		
**Platelets/mm** ^3^						
178+	0	0				
6-177	+0.75	+5	**X**	+5		
**Total individual score**				+21	37.4%	>20% High
**(B) Propensity score relative to simplified score**						
**Simplified score category**				**Estimated propensity to fail REG**		
Low (0-8)				0-5%		
Intermediate (9-16)				6-19%		
High (17+)				20%+		
**(C)**						
The exact formula to calculate the propensity score for a participant (i)						
PS(i) = Num(i)/Den(i)						
Where						
Num(i) = exp (−3.9773 + 1.0209^*^age + 0.5066^*^LDH + 1.1849^*^PaO2/FiO2 ratio + 0.7505^*^PLT)						
Den(i) = 1 + exp (−3.9773 + 1.0209^*^age+0.5066^*^LDH+1.1849^*^PaO2/FiO2 ratio+0.7505^*^PLT)						
In the example of the virtual participant above, as CRP is below the median, it cancels out:						
Num(i) = exp (-3.9773 + 1.0209 + 0.5066 + 1.1849)= 0.59;						
Den (i) = 1 + 0.59 = 1.59; PS= 0.59/1.59= 37.4%						

^$^A virtual fully vaccinated participant, who is aged 62+ with LDH above the median (risk factor) and PaO2/FiO2 ratio (risk factor) and platelets (risk factor) below the median, has an individual score of +21 (i.e., classified as high) which corresponds to an exact estimated propensity to fail regeneron of 37.4%.

## Discussion

To our knowledge, this study can be considered the largest real-world analysis evaluating the effectiveness of CAS/IMV on mortality and MV in subjects with severe COVID-19 at hospital admission. The main result is that, in our setting, where 80% tested SARS CoV-2 negative and approximately 20% of the study population received vaccination for SARS-CoV-2, the risk of day 28 MV/death remained high. Indeed, our cohort exhibited a mortality rate of 6%, with 12% of the patients experiencing the primary end-point MV/death. However, these estimates are much lower than those observed in the RECOVERY trial (24% by day 28 in seronegative patients receiving CAS/IMV vs. 30% of the controls receiving usual care). In contrast, our estimates are similar to those of a recently published study conducted in the USA, which reported a mortality rate of 6% among seronegative patients treated with CAS/IMV compared to 15% of those receiving placebo (15). This difference in the mortality rate across studies is not unexpected and might be explained by the period in which these studies were conducted. Indeed, the randomized studies were conducted between June 2020 and May 2021, before the start of the COVID-19 vaccination campaign. Given the rapid evolution of SARS-CoV-2, changes in virus pathogenicity, and variations in the severity of the conditions among patients admitted to the hospital, even a difference of just 1 month could determine a significant difference in mortality rates. Furthermore, patients enrolled in our study had a lower disease severity at entry and were hospitalized, possibly benefiting from a larger number of drugs recommended in the guidelines. Finally, we cannot rule out that our cohort represents a selected population of individuals who survived long enough after hospital admission to be treated with CAS/IMV.

Contrary to what was previously described, only cardiac insufficiency [and not diabetes or hypertension, for example (19–22)] showed an association with the risk of outcomes after controlling for sex and age. However, this association did not persist after further controlling for other factors included in the score.

The patients who underwent MV or died and the controls differed significantly in age, PaO^2^/FiO^2^ ratio, LDH, and platelet count, all factors that characterized severe COVID-19 since the beginning of the pandemic and were included in similar predictive algorithms. However, it was important to confirm that they are still strong predictors of outcomes in our specific setting.

Prediction accuracy of the derived score was high (84% in the toughest external validation) and did not vary by the endpoint chosen. If anything, the score derived on the composite endpoint was even more predictive when applied to the secondary endpoint of death alone. The score also showed good calibration, a metric which is often neglected when evaluating the performance of a score in addition to discrimination (23). A well-discriminating model similar to our model (i.e., with a *c-statistic* >0.8) may be useless if the decision threshold for clinical decisions is outside the range of predictions provided by the model. Importantly, good calibration was retained in the test set.

Our study has several limitations; first, CAS/IMV are not currently used in clinical practice as they are considered to be not active against the new omicron VoC and therefore it is unlikely that the score could be used in clinical practice for this particular combination. However, monoclonal antibodies are still in development and are tailored to the fast-evolving virus. Some of these new compounds might be re-introduced for the treatment of hospitalized patients as well. Second, it is a retrospective cohort; therefore, data collection was not complete for some biomarkers, and we performed a complete case analysis excluding those with missing laboratory data. Third, we constructed a simple model based on a linear predictor. We cannot rule out that a full machine learning approach with a larger number of variables as well as their interactions could have led to better values for the AUC in validation and test. For example, the score does not account for time-varying data including medical interventions received post hospitalization. Fourth, the sample size of the test cohort was relatively small including approximately 150 participants. However, the AUC in external validation test for both endpoints remained >80% which is considerably above the minimum for a good discrimination.

The main strength of our analysis is that it is the first score derived for hospitalized patients treated with CAS/IMV. In addition, it is very easy to use in practice, uses a small number of routinely collected features, and is calculable without having to input patients' data into a website or a similar graphic user interface (24).

In conclusion, although CAS/IMV is no longer routinely used in clinical practice, our newly developed score might help in identifying at hospital admission which patients are not likely to benefit from monoclonal antibodies and may require alternative interventions.

## Data availability statement

The raw data supporting the conclusions of this article will be made available by the authors, without undue reservation.

## Ethics statement

The studies involving humans were approved by the Institutional Ethics Committee of Area Vasta Emilia Nord. The studies were conducted in accordance with the local legislation and institutional requirements. The Ethics Committee/institutional review board waived the requirement of written informed consent for participation from the participants or the participants' legal guardians/next of kin because Due to the observational nature of the study, written informed consent was not required.

## Author contributions

AC-L: Conceptualization, Methodology, Supervision, Writing – original draft, Writing – review & editing, Formal analysis. VB: Writing – original draft. SR: Writing – original draft. BM: Writing – original draft. AF: Writing – original draft. CD: Writing – original draft. FB: Writing – original draft. PC: Writing – original draft. PM: Writing – original draft. CTo: Writing – original draft. AC: Writing – original draft. GC: Writing – original draft. MB: Writing – original draft. CTa: Writing – original draft. GP: Writing – original draft. SC: Writing – original draft. AG: Writing – original draft. GM: Data curation, Writing – original draft. ML: Writing – original draft. LC: Writing – original draft. CS: Writing – original draft. AD'A: Writing – original draft. VM: Writing – original draft. GG: Data curation, Writing – original draft. EF: Data curation, Writing – review & editing. MM: Writing – original draft. LS: Writing – review & editing. AA: Writing – original draft. EN: Writing – review & editing. CM: Writing – review & editing, Conceptualization, Methodology, Supervision, Writing – original draft.
