# Supplementary material for: Development and validation of a prediction score for failure to casirivimab/imdevimab in hospitalized patients with COVID-19 pneumonia
Source: Front Med (Lausanne). 2024 Mar 11;11:1293431. doi: 10.3389/fmed.2024.1293431 (PMC10961453; doi:10.3389/fmed.2024.1293431)
Supplement: Supplementary file 1 [file Table_1.DOCX]

**Supplementary Material**

**Table S1. Main characteristics of the participants included in the test set by event/event-free status.**

|  | **Event/event-free status (test set)** | | | |
| --- | --- | --- | --- | --- |
| **Markers** | **MV/death** | **Event-free** | **p-value^*^** | **Total** |
|  | N= 24 | N= 133 |  | N= 157 |
| ***Characteristics*** |  |  |  |  |
| Female, n(%) | 11 (45.8%) | 59 (44.4%) | 0.895 | 70 (44.6%) |
| Age, years mean (SD) | 81 (15) | 66 (17) | <.001 | 69 (18) |
| Charlson Index, mean (SD) | 2 (2) | 1 (2) | 0.024 | 1 (2) |
| BMI, mean (SD) | 28 (6) | 28 (6) | 0.788 | 28 (6) |
| Admitted in 2021, n(%) | 19 (79.2%) | 115 (86.5%) | 0.711 | 134 (85.4%) |
| ***Po2/Fo2, mean (SD)*** |  |  |  |  |
| Baseline | 0.5 (0.5) | 0.5 (0.5) | 0.023 | 0.5 (0.5) |
| ***White cells, mean (SD)*** |  |  |  |  |
| Baseline | 8149 (4957) | 6543 (3512) | 0.056 | 6790 (3796) |
| ***IL-6, pg/ml mean (SD)*** |  |  |  |  |
| Baseline | 98.7 (66.4) | 40.3 (38.4) | 0.030 | 46.8 (44.6) |
| ***LDH, mg/dl mean (SD)*** |  |  |  |  |
| Baseline | 325.9 (129.1) | 266.8 (130.7) | 0.043 | 275.8 (131.8) |
| ***hsCRP, mg/dl mean (SD)*** |  |  |  |  |
| Baseline | 3.5 (5.8) | 2.3 (3.7) | 0.171 | 2.5 (4.1) |
| ***Tot Lymphocytes, mg/dl mean (SD)*** |  |  |  |  |
| Baseline | 991.3 (592.1) | 1738 (4532) | 0.423 | 1624 (4183) |
| ***Platelets, mg/dl mean (SD)*** |  |  |  |  |
| Baseline | 165.3 (73.4) | 193.7 (103.4) | 0.199 | 189.4 (99.7) |
| ^*^Chi2 for gender and unpaired t-test | | | | |

N: number; LDH: lactate dehydrogenase; IL-6: interleukin-6; BMI: body mass index; MV: mechanical ventilation; SD: standard deviation;

**Figure S1. Calibration plot**

**Table S2. Odds ratio of death from fitting a logistic regression model (Training set)**

|  | **OR of MV/death (Training dataset)** | | | | | |
| --- | --- | --- | --- | --- | --- | --- |
|  | **Unadjusted OR (95% CI)** | **p-value** | **Adjusted1^*^ OR (95% CI)** | **p-value** | **Adjusted2^&^ OR (95% CI)** | **p-value** |
| ***P/F, per log10 lower*** |  |  |  |  |  |  |
| Baseline | 42.33 (4.88, 366.9) | <.001 | 30.97 (2.91, 329.7) | 0.004 | 38.77 (1.85, 812.8) | 0.018 |
| ***IL-6, per log10 mg/dl higher*** |  |  |  |  |  |  |
| Baseline | 1.88 (1.00, 3.52) | 0.050 | 2.49 (1.11, 5.56) | 0.026 |  |  |
| ***LDH, per log10 mg/dl higher*** |  |  |  |  |  |  |
| Baseline | 3.36 (0.73, 15.52) | 0.120 | 6.84 (1.18, 39.63) | 0.032 | 2.63 (0.31, 22.54) | 0.377 |
| ***Platelets, per log10 mg/dl lower*** |  |  |  |  |  |  |
| Baseline | 12.79 (2.58, 63.35) | 0.002 | 10.52 (1.74, 63.60) | 0.010 | 14.09 (1.72, 115.2) | 0.014 |
| ***White Cells, per log10 higher*** |  |  |  |  |  |  |
| Baseline | 0.79 (0.17, 3.57) | 0.759 | 0.70 (0.15, 3.28) | 0.648 |  |  |
| ***Lynphocytes, per log10 higher*** |  |  |  |  |  |  |
| Baseline | 4.60 (1.15, 18.29) | 0.030 | 2.46 (0.57, 10.57) | 0.228 | 1.05 (0.23, 4.75) | 0.950 |
| ***hsCRP, per log10 higher*** |  |  |  |  |  |  |
| Baseline | 1.01 (0.70, 1.45) | 0.969 | 1.15 (0.70, 1.89) | 0.570 | 1.09 (0.64, 1.87) | 0.750 |
| ***Age, per 10 years older*** | 2.33 (1.69, 3.19) | <.001 |  |  |  |  |
| ***Charlson Index, per 1 unit higher*** | 1.19 (1.02, 1.40) | 0.029 |  |  |  |  |
| ***Female vs. male*** | 0.74 (0.33, 1.64) | 0.456 |  |  |  |  |
| ***Cardiac insufficiency*** | 3.74 (1.49, 9.37) | 0.005 | 1.55 (0.58, 4.20) | 0.005 | 0.84 (0.26, 2.77) | 0.781 |
| ***Renal disease*** | 3.17 (1.12, 8.95) | 0.029 | 1.01 (0.32, 3.19) | 0.029 | 0.89 (0.24, 3.34) | 0.860 |
| ***Vasculopathy*** | 3.11 (1.00, 9.71) | 0.051 | 1.31 (0.38, 4.54) | 0.051 | 1.70 (0.44, 6.57) | 0.440 |
| ***BMI, >30 vs. <=30*** | 0.55 (0.18, 1.65) | 0.288 |  |  |  |  |
| ***Year of hospital admission, 2022 vs. 2023*** | 0.28 (0.12, 0.66) | 0.004 | 0.45 (0.18, 1.17) | 0.100 | 0.42 (0.13, 1.35) | 0.144 |
| ***At least 2 doses of vaccine, yes vs. no*** | 1.48 (0.63, 3.49) | 0.370 |  |  |  |  |
| ***SARS-CoV-2 serology, pos vs. neg*** | 1.48 (0.60, 3.66) | 0.398 |  |  |  |  |
| ^*^adjusted for gender and age | | | | | | |
| ^&^adjusted for gender age and other markers shown in Table | | | | | | |

CRP: C-reactive protein; BMI: body mass index; IL-6: interleukin-6; MV: mechanical ventilation.

**Figure S2. Internal cross-validation results. ROC of AUC endpoint death**

**Figure S3. External cross-validation results. ROC of AUC endpoint death**

**Table S3.** Contributing centres

| **Hospital** | **N** | **% of set** |
| --- | --- | --- |
| **Training set** | | |
| **INMI Spallanzani Roma** | 176 | 37% |
| **Latina Hospital** | 30 | 6.3% |
| **Unimore Modena** | 78 | 16.3% |
| **Padova Hospital** | 63 | 13.1% |
| **Pescara Hospital** | 2 | 0.4% |
| **University of Tor Vergata Roma** | 54 | 11.3% |
| **San Remo Hospital** | 14 | 2.9% |
| **Trapani Hospital** | 5 | 1.0% |
| **Udine Hospital** | 58 | 21.1% |
| Total training | 480 | 100.0% |
| **Test set** | | |
| **Catanzaro Hospital** | 31 | 19.8% |
| **San Paolo Hospital Milano** | 70 | 44.6% |
| **La Sapienza University Roma** | 56 | 35.7% |
| Total test | 157 | 100.0% |
